# How Does Artificial Intelligence Align With Person‐Centred Principles in Mental Health Nursing? A Scoping Review

**DOI:** 10.1111/inm.70282

**Published:** 2026-06-10

**Authors:** Carmel Bond, Adrianna Lorraine Watson, Graeme D. Smith, Helen Aveyard, Debra Jackson

**Affiliations:** ^1^ Department of Nursing and Midwifery, School of Health and Social Care Sheffield Hallam University Sheffield UK; ^2^ College of Nursing Brigham Young University Provo Utah USA; ^3^ S.K. Yee School of Health Sciences, Saint Francis University Tseung Kwan O Hong Kong; ^4^ Oxford Brookes University Oxford UK; ^5^ School of Nursing University of Sydney Sydney New South Wales Australia

**Keywords:** artificial intelligence, mental health, nursing, person‐centred care, psychiatric nursing

## Abstract

Artificial intelligence is increasingly used in mental health nursing, yet its alignment with person‐centred care remains underexplored. This scoping review examines the extent to which artificial intelligence applications in mental health nursing align with person‐centred principles, and where tensions and risk emerge. A systematic search of three electronic databases identified studies published since 2018. Data were charted for study characteristics, artificial intelligence modalities, person‐centred care concepts addressed, and research gaps. Findings show growing interest in technology‐enabled care delivery, monitoring, and decision support in mental health settings, with varying degrees of attention to person‐centred values such as empathy, shared decision‐making, dignity and therapeutic alliances. However, considerable gaps remain regarding ethical integration, digital therapeutic relationships, trust, surveillance and the measurement of person‐centred care outcomes. This review maps the current evidence base and highlights critical gaps in understanding how artificial intelligence reshapes therapeutic relationships, professional roles and power dynamics in mental health nursing. Future research is needed to ensure that the adoption of artificial intelligence is not only safe and effective, but also ethically grounded and aligned with the humanistic foundations of person‐centred mental health nursing.

## 1 Introduction

Healthcare systems worldwide are being transformed by artificial intelligence (AI), which aims to enhance patient outcomes and improve healthcare delivery (Bekbolatova et al. 2024; Milasan and Scott‐Purdy 2025). From predictive analytics designed to optimise resource allocation to conversational agents supporting patient self‐management, AI promises efficiency gains, personalised interventions, and expanded access amid growing workforce pressures and increasing service demands (Davenport and Kalakota 2019). Within nursing, these technologies are no longer speculative but embedded in everyday practice (Jackson 2026), reshaping clinical documentation and treatment planning; prompting new forms of human‐machine collaboration (Watson et al. 2025; Chen and Lee 2025). While these developments are often framed as neutral or beneficial innovations, they also reflect broader institutional priorities around efficiency, standardisation, and scalability that have implications for how care is organised and experienced in practice (McCormack 2025; von Arx 2023). Mental health nursing occupies a distinct position within this transformation, grounded in relational approaches that prioritise empathy, therapeutic engagement, and recovery‐oriented practice over the procedural efficiencies that often characterise other areas of nursing (Bond, Painter, et al. 2025; Milasan and Scott‐Purdy 2025). Holistic, person‐centred care, defined by respect for individuality, shared decision making, and holistic needs assessment, forms the ethical foundation across all fields of nursing (McCormack and McCance 2021). However, while mental health nursing has, historically, faced barriers in establishing a clearly defined professional identity, the cultivation of therapeutic relationships as a vehicle for recovery is fundamental to mental health nursing practice (Bond et al. 2022). Compassion is also central to mental health nursing and is characterised by relational communication and an active effort to understand each patient's individual experiences (Bond, Usher, et al. 2025). These humanistic principles may shape how AI is understood and implemented within mental health nursing practice, where care is inherently relational, and context specific (Bond 2025).

AI applications with relevance to mental health nursing are diverse and expanding; digital phenotyping for assessment, relapse prediction algorithms analysing speech patterns, computer vision for facial emotion recognition, resource optimisation models, and staff/patient perceptions of chatbots (Milasan and Scott‐Purdy 2025). App‐based AI tools delivering cognitive behavioural interventions for anxiety, stress, and depression are increasingly common, extending care beyond clinical encounters to support patient self‐management, and allowing professionals to monitor emotional states and distress remotely and continuously (Olawade et al. 2024). These innovations align with policy priorities for digital mental health expansion while addressing global service gaps (NHS Long Term Plan 2019; WHO 2024). At the same time, these technologies create new ways of observing, recording, and interpreting distress within clinical practice (Alowais et al. 2023). The growing use of AI introduces new considerations for how mental health nurses communicate with patients across both digital and face‐to‐face interactions (Maqsood et al. 2024). This means mental health care supported by AI has immediate implications for assessment, monitoring, treatment, and nurses' ability to uphold person‐centred, recovery‐orientated principles (Nilsen et al. 2022).

Previous scholarship has confirmed the pivotal role of empathy across therapeutic phases: perspective‐taking builds early alliances, while empathic concern sustains working relationships through distress and mental health crises (Moreno‐Poyato et al. 2021). Yet, the technical capabilities of AI stand in contrast to mental health nursing's humanistic, interpersonal core (Bond, Usher, et al. 2025). Unlike diagnostic imaging or vital signs analytics in physical health, mental health practice demands nuanced relational judgment, empathy, compassion, and ethical navigation of risk, coercion, and existential distress: forms of interpretive and moral labour that may be beyond the current scope of AI (Jackson 2025, 2026; McCormack 2025). In mental health settings, nurses often work to balance safety, autonomy, and trust within complex therapeutic relationships (Bond 2025).

So far, empirical work has focussed on the procedural benefits of using AI for aiding diagnosis or workflow optimisation in the general nursing environment (Watson et al. 2025), neglecting the recovery‐oriented approach that underpins contemporary mental health nursing (Santos et al. 2018). A strong focus on efficiency and standardisation may create tensions with the relational priorities of person‐centred mental health nursing. Moreover, previous reviews have considered the various applications of AI in mental health nursing (Milasan and Scott‐Purdy 2025); yet they fall short of systematically evaluating alignment with person‐centred care frameworks (McCormack and McCance 2021), thereby limiting critical insight into how these technologies interact with the values, relationships, and power dynamics that shape nursing practice. As such, the current review aimed to examine AI's compatibility with person‐centred principles in mental health nursing. The review question was: *How do applications of artificial intelligence in mental health nursing practice align with person‐centred care principles, and what are the associated benefits, challenges, and ethical considerations that arise from this alignment?*

## 2 Aims

To examine and map the current literature on AI applications relevant to mental health nursing practice, with a focus on how these technologies align with the person‐centred care principles and the benefits, challenges and ethical tensions this alignment generates.

## 3 Methods

This review followed Arksey and O'Malley's (2005) five‐stage framework (Levac et al. 2010). This approach was selected to describe and map the key areas of published research concerning artificial intelligence in mental health nursing, identifying key concepts and the range of available evidence (Aveyard et al. 2021). Reporting followed the Preferred Reporting Items for Systematic Reviews and Meta‐Analyses extension for Scoping Reviews (PRISMA‐ScR) (Tricco et al. 2016) and was informed by the latest methodological guidance for scoping reviews (Peters et al. 2020). Unlike systematic reviews, which aim to synthesise findings to answer tightly focused questions, scoping reviews address broader or more exploratory topics and include diverse study designs without formal quality appraisal as a central aim. This flexibility makes the scoping review method appropriate for areas characterised by emerging evidence (Hadie 2024; Tricco et al. 2016).

### 3.1 Inclusion Criteria

The review followed the participants, concept, context framework (Peters et al. 2020; Michalowski et al. 2025).

**Table:** 

	Inclusion	Exclusion
Participants	Mental health nurses	Studies not involving mental health nurses in clinical practice; student nurses
Concept	AI applications	No explicit artificial intelligence applications; general digital health only
Context	Clinical Context—Mental health nursing	Physical health conditions only; medical/treatment‐focused without nursing relevance
Types of Sources	Empirical studies—English	Editorials, opinion pieces, conference abstracts, gey literature, literature reviews

### 3.2 Search Strategy

A comprehensive, iterative, search strategy was conducted across Scopus, CINAHL, and APA PsycInfo. The choice to include literature from the last 7 years reflects the rapid growth of AI technologies, particularly following the introduction of ChatGPT (Wu et al. 2023), ensuring that the review captures the contemporary context relevant to the research question.

#### 3.2.1 Stage One

The first stage involved a pilot search 30 of keywords and controlled vocabulary terms. Keywords representing each central concept were combined using the Boolean operator OR to create sets of synonyms and related terms. These sets were then linked with the operator AND to retrieve studies addressing all facets of the research question simultaneously. We focussed on words related to the participants ‘mental health nurses’, the concept of AI, and relevant synonyms. The Boolean operator NOT was applied to exclude literature pertaining to student nurses. The search strategy was iteratively refined to enhance the relevance and precision of results. Techniques such as truncation ‘()’ and the use of quotation marks to enclose phrases were employed to capture variations in terminology. Controlled vocabularies, including MeSH terms and CINAHL subject headings, were integrated where appropriate to increase search sensitivity.

#### 3.2.2 Stage Two

A comprehensive search was conducted in Scopus, CINAHL, and APA PsycINFO, with the most recent search run on 7 July 2025. The strategy combined three concept blocks (mental health nursing, artificial intelligence, and related machine learning terms), using Boolean operators and an exclusion for student populations. Searches were limited to peerreviewed journal articles, humans, English language, and the period 1 July 2018–1 July 2025. An example Scopus strategy was: TITLE‐ABS‐KEY(‘menta‐l health nurs*’ OR ‘psychiatric nurs*’) AND TITLE‐ABS‐KEY(‘artificial intelligence’ OR AI OR ‘machine learning’ OR ‘deep learning’ OR ChatGPT). References lists were manually searched in stage three. The final deduplicated total across all three databases was 172 records prior to screening. The full database‐specific search strategies are provided to support reproducibility (Appendix A).

### 3.3 Selection of Studies

Retrieved records were screened against inclusion/exclusion criteria using Covidence systematic review software (Veritas Health Innovation, Melbourne, Australia; www.covidence.org). Screening comprised two phases: title/abstract review followed by full‐text assessment of potentially eligible studies.

#### 3.3.1 Exclusion Criteria

- Unrelated to mental health nursing/clinical workflow
- Editorials, comment pieces, review articles
- No explicit AI content
- Student‐focused or non‐clinical education only
- Medical/treatment‐focused (unless nursing‐relevant)
- Non‐English
- Physical health only or related to medical/treatment

Following PRISMA‐ScR guidance (Tricco et al. 2016), grey literature was considered but excluded due to the scoping review aims of describing and mapping current evidence on AI applications in mental health nursing, and relevance to clinical workflow (Peters et al. 2020).

**FIGURE 1:**
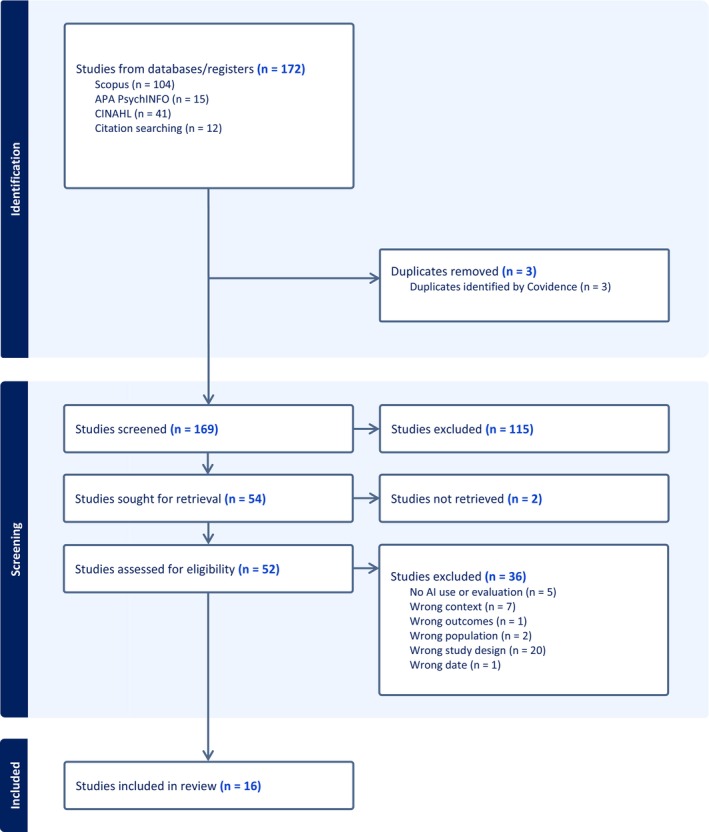
PRISMA diagram of study selection process.

Selection workflow is summarised in Figure 1.

Although the review primarily included studies published from 2018 onwards to focus on recent developments, one study from 2014 (Louie et al. 2014) was retained (but not included in the analysis) due to its foundational relevance to the acceptance of socially assistive robots in nursing.

### 3.4 Data Extraction

Each full‐text article was independently assessed by two reviewers (CB and AW), with any discrepancies settled by consultation with a third reviewer (DJ). Data were extracted using a structured template capturing author, year, location, design, population, contextual details, findings, and individual and organisational implications. An initial textual synthesis approach was employed, summarising and bringing together results across studies to highlight recurring patterns, contrasts, and evidence gaps. A summary table (Table 1) was developed to clearly present and compare essential details from each included study.

**TABLE 1:** Data matrix.

Author(s)/Year	Aim of study	Geographical location of study' as one study states 'Europe'	Participants	Study design	Summary of findings relevant to the review
Wand et al. (2022)	To explore the perspectives of MHNs, consumers, and allied health staff on the current status and future direction of mental health nursing	Australia	Total of 11 participants (three groups). One group consisted of five senior clinical MNHs; A second was held with three consumer representatives, and a third focus group with three allied health professions	Qualitative/Thematic Analysis	MHNs: value therapeutic, person‐centred, holistic, and empowering roles; felt undervalued and constrained by admin/biomedical focus Consumers: criticized paternalism, medication dominance; advocated ‘nothing about us without us,’ collaborative documentation, peer support, and therapeutic connection. Allied health: noted task‐driven inpatient nursing, burnout issues; called for more therapeutic engagement, social determinant focus, and trauma‐informed care Summary: AI can align with person‐centred care if it reduces admin burden, supports shared decision‐making, collaborative records, and empowerment. Risks if used for surveillance, control, or reinforcing biomedical models
Imkome et al. (2025)	Study explored the feasibility and potential effectiveness of the Ai‐Aun chatbot in enhancing mental health among older adults in Thailand	Thailand	Adults aged 60 and older residing in the Bang‐kra‐beo sub‐district, Pathum Thani province, Thailand	Quasi‐experimental design, *N* = 44 older adults (22 experimental, 22 control). Experimental group used Ai‐Aun chatbot for 15 days	Significant improvement in mental health scores in the chatbot group versus control Design emphasised natural language, trust, usability, and personalisation Privacy safeguards and transparency increased trust and uptake, which demonstrates importance of personalisation, CBT integration, and safeguarding ethics/privacy
Nashwan et al. (2023)	Addresses AI's ethical and responsible use in mental health nursing, emphasizing patient privacy, data security, and the balance between human interaction and AI tools	Qatar	N/A	Narrative review	Highlights AI's potential to align with person‐centred care if implemented responsibly (supporting decision‐making, personalisation, and accessibility) while cautioning against risks to trust, ethics, and human connection
Doğu et al. (2025)	Study investigated the usability of artificial intelligence and machine learning techniques to predict individuals' levels of self‐sensitivity and compassion	Türkiye	307 students	Quantitative: Regression Model/cross‐sectional, questionnaire	AI models can accurately predict self‐sensitivity and compassion scores, supporting efficient assessment of psychosocial traits linked to resilience and care quality Study shows that AI can support person‐centred care by monitoring/assessing relational qualities but must complement (not replace) human empathy and relational judgement.
Barrera et al. (2020)	To describe the process of introducing artificial intelligence (‘digitally assisted nursing observations’) in an acute mental health inpatient ward, to enable staff to carry out the hourly and the 15minutes observations, minimising disruption of patients’ sleep while maintaining their safety	UK	Observations estimated to cover 755 patients	Pilot study	AI‐enabled optical sensors accurately recorded safety/vital signs during nightly observations, reduced sleep disturbance, and were well‐accepted by staff, patients, and relatives; preliminary evidence suggested possible improvements in sleep without increasing medication use or admission length Study demonstrates AI's potential to align with person‐centred care by enhancing safety while preserving rest, autonomy, and dignity; highlights need for ethical oversight and further robust evaluation.
Abou Hashish (2025)	To explore and define the concept of digital empathy and its determinants within the context of nursing practice	Saudi Arabia	N/A	Review/concept analysis A total of 52 sources were reviewed, comprising 46 research articles, four books, and two web pages, published between 2000 and 2024	Study identifies digital empathy as essential for telehealth care; attributes include authenticity, trust‑building, responsiveness, adaptability, emotional engagement, cultural sensitivity, and technological proficiency Antecedents: digital literacy, emotional intelligence, supportive environments. Consequences: improved patient satisfaction, trust, adherence, nurse well‑being, and organisational retention AI/telehealth tools must be designed to enhance digital empathy and support genuine, person‑centred interactions; risks arise if technology prioritises efficiency over authenticity and emotional connection
Alves et al. (2025)	Study developed, validated and applied a machine learning model to estimate PHQ‐9 scores for MDD patients using relevant notes from electronic medical records (EMR)	USA	N/A	Model development including sources on over 3.5 million patients receiving treatment from over 9000 mental health professionals across 2500 clinics in all 50 United States	Study suggests that AI can strengthen person‑centred monitoring by enhancing availability and equity in symptom measurement, supporting proactive, collaborative decisions For alignment with person‑centred principles, AI outputs must be integrated as supportive tools, not replacements for patient voice and lived experience
Garcia et al. (2025)	To explore whether responses to open‐ended questions, quantified using AI, provide additional value in measuring intervention outcomes compared to traditional rating scales	Sweden	Swedish adolescents (*N* = 44) who received Internet‐based Cognitive Behavioral Therapy (ICBT) for eight weeks	Mixed methods—survey followed by open questions	Study suggests that AI can complement traditional scales by embedding patient narratives into outcome measurement, strengthening alignment with person‑centred approaches Adds ecological validity and holistic understanding, provided it is used to enrich (not replace) patient reporting
Hu et al. (2023)	To estimate the violence rate for psychiatric inpatients and establish a predictive model for violence in psychiatric inpatients	Singapore	N/A	Structured and unstructured data from Chinese nursing electronic medical records (EMRs) for the violence prediction Descriptive statistics were performed	Findings indicated that AI can help anticipate risk to improve ward safety, but alignment with person‑centred principles requires ethical safeguards, transparency, shared decision‑making, and emphasis on dignity to avoid coercion or reinforcing power imbalances AI could improve safety and tailored care planning in psychiatric wards. However, risks of labeling, stigmatization and undermining trust if used as a surveillance tool rather than supporting therapeutic engagement
Kenigsberg et al. (2019)	To analyse how assistive technologies can address the capabilities of people with dementia, on the basis of their needs	Europe	20 participants from the fields of cognitive neurosciences, computer sciences, economics, ergonomics, geriatrics, neurology, occupational health, philosophy, psychiatry, public health, cognitive psychology, social psychology, social policy, sociology, and gerontology	Multiple methods	Assistive technology can help people with dementia in several ways: (1) Co‐production and shared decision‐making with people living with dementia; (2) Technology tailored to individual needs, preferences, and capabilities; (3) Supports autonomy, dignity, social inclusion, and meaningful engagement; and (4) Encourages ethical, equitable, and user‐friendly access and use Work serves as a model for embedding person‐centred values in AI‐enabled dementia care, stressing user autonomy, ethical safeguards, and ongoing user involvement
Palmer et al. (2025)	To evaluate the engagement, clinical effectiveness, acceptability, and safety of a digital anxiety intervention that combined an AI‐powered conversational agent with human clinical support, compared with waiting‐list controls and human‐delivered CBT (typed and face‐to‐face)	UK	Prospective participants (*N* = 299) were recruited from the National Health Service (NHS) or social media in the United Kingdom mild to severe generalized anxiety disorder (GAD‐7 > 7; PHQ‐9 < 16)/*N* = 299 adults/aged 18–75, majority of sample female (80.3%) and White (89%).	Non‐randomised experimental study	Evidence‐based, human‐supported digital program for adults with mild to severe anxiety produced a large, clinically meaningful reduction in anxiety symptoms and significantly reduced clinician time compared to traditional care Study findings underscore the potential of combining AI‐driven digital programs with human support to expand access to effective, scalable mental health care while preserving treatment quality and supporting person‐centred principles through tailored program content and ongoing human interaction
Qiao et al. (2025)	To identify predictive variables of suicide risk in patients with schizophrenia using machine learning models, in order to improve clinical suicide risk assessment and prevention strategies	Chengdu, China	Mental Health Center, West China Hospital, Sichuan University/131 inpatients with schizophrenia (ages 16–35)/65 male, 66 female; mostly Han Chinese	Cross Sectional	Findings suggest integrating biological markers with psychosocial and nursing assessment tools may improve suicide risk stratification and support tailored interventions in schizophrenia care
Rogan et al. (2024)	To explore mental health professionals' views on using digital devices to passively collect data and apply machine learning in mental healthcare, as well as the potential barriers and facilitators to their implementation in practice	UK	Multidisciplinary secondary mental health staff/15 participants/included clinical psychologists, psychiatrists, mental health nurses, occupational therapists, specialist practitioners, and consultants	Qualitative (interviews)	Study highlights the potential of AI‐powered passive sensing to augment care but stresses ethical, privacy, and relational considerations in deployment Findings demonstrate that successful AI integration requires addressing both technological capabilities and socio‐emotional factors to align with person‐centred care ideals
Tuncer and Duman (2025)	To explore psychiatric nurses' perspectives regarding AI applications in supporting both direct and indirect care processes	Türkiye	Postgraduate students or graduates (Master's or PhD) in psychiatric nursing/28/Roles included academics, hospital psychiatric nurses, community mental health nurses, intensive care nurses	Descriptive qualitative	Nurses consistently emphasised that AI cannot replace human qualities such as empathy, face‐to‐face interaction, and the ability to manage complex psychiatric symptoms but acknowledge AI's supportive role in providing rapid access to information, assisting with care planning, and offering simulation‐based training for psychiatric nursing skills Concerns about data security, trust, risk of patient harm, professional devaluation, and potential erosion of educators’ roles or research skills underscored the caution with which nurses view AI's place in psychiatric nursing
Wang and Li (2024)	To compare the effects of AI chatbots (ChatGPT 3.0) with traditional mindfulness therapy on loneliness and depression in older adults, and to explore the potential of AI as a complementary or alternative mental health support tool	China	Inclusion criteria: adults over 60, able to communicate in Mandarin, normal cognitive function (MMSE > 26), with basic mobility and communication skills/4 ChatGPT and 8 mindfulness group completed/	Non‐randomised experimental study	Participants valued companionship and relaxation but noted that ChatGPT felt robotic, and mindfulness required effort to sustain Study suggests ChatGPT may offer comparable short‐term emotional benefits to mindfulness, though conclusions are limited by the small, unbalanced sample and short duration
Wilson et al. (2024)	To (1) explore issues around maintaining quality of life and (2) obtain greater understanding of collaboration and shared decision making between people with dementia and their ‘informal carers’	Ireland	Individuals with a diagnosis of pre‐dementia or early‐stage dementia AND informal carers AND health professionals/5 people living with dementia/4 informal carers/5 health professionals/interviews in homes lasting ∼60 min	Qualitative (interviews)	Study highlights how AI and technology design for dementia care should focus on promoting autonomy, preventing learned helplessness, and enabling collaborative care planning Findings emphasise the mental health benefits of meaningful activity engagement and reduced caregiver burden, pointing to AI‐supported personalisation and adaptive systems as critical to future interventions in mental health nursing

### 3.5 Data Analysis and Synthesis

Following data extraction, a qualitative synthesis method was used to integrate findings across studies. This approach involves summarising and interpreting patterns, similarities, and differences within the evidence base (Pollock et al. 2024). This approach is well‐suited for scoping reviews as it allows for a descriptive overview of complex and heterogeneous literature (Campbell et al. 2020).

The evidence was mapped thematically, grouping studies according to key concepts, populations, or types of interventions to provide a structured summary of the field (Peters et al. 2020). This mapping facilitates identification of prevalent themes as well as gaps in the current knowledge (Mak and Thomas 2022). Thematic or categorical summaries were developed to highlight areas of concentration and areas where further research is needed. Together, these methods provide a comprehensive overview of the extent, range, and nature of research evidence related to the use of artificial intelligence in mental health nursing.

## 4 Results

### 4.1 Characteristics of Included Studies

The included studies collectively examined a broad range of aims related to the integration and implications of AI within mental health nursing and associated care contexts. Ethical considerations, clinical effectiveness, user and professional perspectives, psychosocial outcomes, and technological innovations were explored across the included studies, although the depth of engagement with person‐centred care principles varied considerably. Various methodological approaches were taken; these included qualitative interviews (Wilson et al. 2024; Rogan et al. 2024; Tuncer and Duman 2025), non‐randomised experimental and mixed‐methods designs (Garcia et al. 2025; Palmer et al. 2025; Wang and Li 2024), cross‐sectional analyses (Hu et al. 2023; Qiao et al. 2025), and descriptive or multiple methods approaches (Kenigsberg et al. 2019).

Populations studied ranged from mental health professionals and patients to older adults and individuals living with dementia, across diverse geographic and care settings. Sample sizes ranged widely, from small qualitative groups, such as five people with dementia (carers and professionals) (Wilson et al. 2024), 15 mental health professionals (Rogan et al. 2024), and 28 psychiatric nurses (Tuncer and Duman 2025), to larger quantitative cohorts, including 299 adults with anxiety (Palmer et al. 2025), 131 inpatients with schizophrenia (Qiao et al. 2025), and 44 adolescents receiving internet‐based cognitive behavioural therapy (Garcia et al. 2025). This variation reflects the heterogeneity of both mental health populations and the contexts in which AI applications are being explored.

Studies were conducted across multiple countries including Australia, Sweden, Singapore, Türkiye, Canada, the UK, China, Ireland, Thailand, and Europe. Populations included adolescents receiving internet‐based cognitive behavioural therapy (Garcia et al. 2025), older adults engaging with AI chatbots (Wang and Li 2024; Imkome et al. 2025), psychiatric inpatients (Hu et al. 2023; Qiao et al. 2025), mental health professionals (Tuncer and Duman 2025; Rogan et al. 2024), and people living with dementia and their carers (Kenigsberg et al. 2019; Wilson et al. 2024). These studies spanned a range of clinical, community, and digital care settings, reflecting the breadth of contexts in which AI is being introduced into mental health nursing practice.

Key thematic focuses included the development and evaluation of AI tools for symptom monitoring, digital empathy, therapeutic support, ethical implementation, safety, and relational care. Collectively, these studies provide a foundation for understanding the multifaceted roles, opportunities, and tensions associated with the use of AI in advancing person‐centred mental health nursing, particularly in relation to person‐centred care.

### 4.2 Key Concepts Identified Across the Studies

**FIGURE 2:**
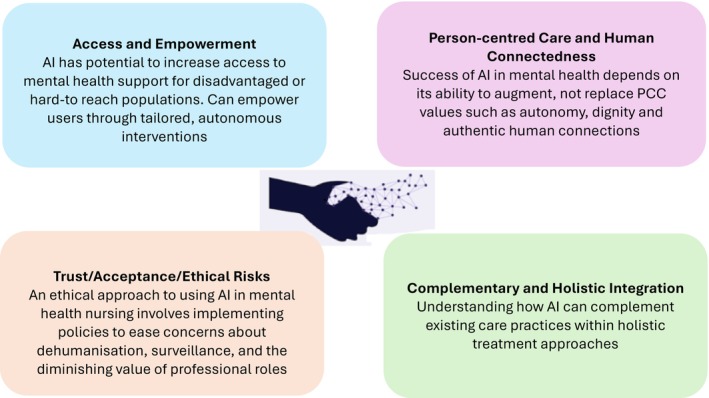
Summary of key concepts mapped to themes.

This section presents a narrative synthesis of the primary themes identified through systematic mapping and analysis of the included studies. It aims to capture the core concepts, patterns, and relationships emerging across the literature on AI in mental health nursing, with particular attention to how these intersect with person‐centred care principles. The identified themes encapsulate the multifaceted roles, implications and tensions associated with AI in mental health nursing and are summarised in Figure 2.

#### 4.2.1 Access and Empowerment

AI was shown to enhance patient empowerment across clinical contexts by fostering therapeutic alliance (Imkome et al. 2025), thereby enabling equitable monitoring without silencing patient voices (Alves et al. 2025), and supporting autonomy through shared decision‐making in dementia care (Kenigsberg et al. 2019; Wilson et al. 2024). AI chatbots expanded access across diverse populations by providing 24/7 support to overcome key barriers—including service shortages affecting both patients and nurses. Wang and Li (2024) demonstrated this through CBT/mindfulness exercises delivered using AI chatbots, thus reducing feelings of loneliness, while Imkome et al. (2025) confirmed improved mental health outcomes using empathetic, user‐friendly chatbots. Together, these studies suggest that AI may support more timely and accessible care in contexts where traditional services are limited. At the same time, access within these studies was largely mediated through digital platforms, raising questions about engagement among populations experiencing vulnerability or digital exclusion.

#### 4.2.2 Person Centred Care and Human Connectedness

Across the included studies, AI was generally positioned as a tool intended to support rather than replace person‐centred aspects of care. For instance, Abou Hashish's (2025) conceptualisation of digital empathy reveals that emotional engagement and trust‐building remain central, requiring compassionate communication and adapting traditional empathy for virtual settings. These findings suggest that while AI may support personalised care processes, limitations remain in relation to emotional depth and relational engagement.

Garcia et al. (2025) demonstrated how AI‐assisted analysis could capture aspects of adolescents' recovery narratives that extended beyond standard rating scales. The findings also highlighted the continuing importance of nurses' relational skills in eliciting and interpreting patient narratives. The included studies consistently emphasised the importance of human connectedness and collaborative care processes. AI could support some aspects of these processes but was not presented as independently capable of sustaining therapeutic relationships. This is exemplified by Wilson et al. (2024), who employed qualitative thematic analysis of interviews/focus groups with early‐stage dementia couples, their families and professionals, identifying shared decision‐making and purpose/identity as essential themes requiring collaborative technology design that balances patient empowerment with carer support—whilst at all times under the relational mediation of the professional nurse.

#### 4.2.3 Trust, Acceptance and Ethical Risks

AI in mental health nursing was associated with issues of trust, transparency, training, and privacy, alongside risks of dehumanisation, surveillance, and professional role change, as reflected across the studies included. Based on qualitative interviews with 28 psychiatric nurses, Tuncer and Duman (2025) reported that AI‐supported systems were perceived to reduce routine workload and enable greater focus on psychosocial care and therapeutic relationships. However, participants emphasised that AI could not replicate empathy, communication, or relational judgement central to mental health nursing practice. These findings may support efficiency‐related aspects of care while contributing less directly to relational and ethical dimensions of practice.

In two studies, AI was used to support clinical risk prediction without replacing nurses' ethical judgement, particularly in relation to violence and suicide (Hu et al. 2023; Qiao et al. 2025). Machine learning models were developed to predict violence among 406 psychiatric inpatients using demographic and clinical variables to prioritise de‐escalation planning (Hu et al. 2023), while suicide prediction algorithms combined nursing assessments with biological markers to trigger early prevention protocols (Qiao et al. 2025). Across these studies, nurses remained central to interpreting risk information within broader relational and clinical contexts. Tensions surrounding trust and ethical use were also evident in relation to passive sensing technologies, which enabled continuous monitoring but raised concerns about privacy and the potential erosion of therapeutic relationships (Rogan et al. 2024). Within this study, these challenges were described as particularly pronounced for patients experiencing paranoia, requiring clinicians to balance technological insight with sensitivity to individual experience. In this context, nurses were positioned as mediators between AI‐generated data and person‐centred care. Overall, the included studies position AI as a supportive tool within mental health nursing, with its use shaped by ongoing negotiation between technological capability, ethical considerations, and the relational requirements of care.

#### 4.2.4 Complementary and Holistic Integration

Across the included studies, AI was generally presented as complementing rather than replacing nurses' observational and relational roles. For example, Barrera et al. (2020) piloted Oxehealth AI sensors for night‐time observations across 755 patient‐nights in acute psychiatric care, achieving 100% agreement with in‐person checks while enhancing patient safety, autonomy, and sleep through minimised disruptions: freeing mental health nurses for therapeutic engagement. These findings suggest that while AI may support safety monitoring, ongoing nursing oversight remains important for maintaining relational aspects of care.

AI can be used to scale evidence‐based interventions without supplanting nurses' clinical relationship, as shown by Palmer et al. (2025) who demonstrated AI conversational agents delivering clinician‐written CBT content to 299 anxiety patients in 1.6 clinician hours versus eight hours traditionally, whilst Alves et al. (2025) developed machine learning reading ePHQ‐9 depression scores from 450 000+ clinician notes; tripling monitoring data availability while accurately distinguishing mild vs. serious cases, requiring careful nursing monitoring to ensure clinical validity. Across these studies, workflow efficiencies associated with AI continued to depend on nurses' ability to interpret and contextualise automated outputs within therapeutic care. These studies demonstrate AI's capacity to eliminate repetitive assessments (Alves et al. 2025), scale CBT delivery (Palmer et al. 2025) and automate safety checks (Barrera et al. 2020) while preserving nurses' indispensable role in therapeutic relationship‐building, essential to person‐centred mental health practice. Overall, the included studies suggested that AI functioned most effectively when integrated alongside, rather than in place of, therapeutic human interaction.

## 5 Discussion

This review aimed to systematically identify, collate, and undertake an initial synthesis of current evidence on the applications of artificial intelligence relevant to mental health nursing practice, with a focus on identifying what we know about the potential benefits, challenges, and ethical considerations impacting person‐centred care delivery (McCormack and McCance 2021).

Studies of chatbot interventions demonstrate potential to improve mental health outcomes and increased engagement among groups who may otherwise face stigma or limited‐service availability (Imkome et al. 2025; Wang and Li 2024). Similarly, machine learning applications that consistently monitor depressive symptoms (Alves et al. 2025), dementia‐focused AI initiatives (Kenigsberg et al. 2019), and dementia co‐design research (Wilson et al. 2024) highlight AI's potential capacity to reduce inequities and deliver more personalised, dignified care at scale. These findings suggest that when AI is user‐centred in design, it can act as a practical extension of person‐centred care values (McCormack and McCance 2021). At the same time, however, the focus on efficiency, immediacy, and large‐scale depersonalisation evident in AI healthcare applications more broadly (Manickam et al. 2022; Rasa 2024; von Arx 2023) raises questions about whether such efficiency‐driven advances genuinely enhance person‐centredness or risk reframing care around speed and optimisation rather than shared meaning and relational depth.

Relational aspects of person‐centred care emerged as one of the most complex areas for AI integration (McCormack and McCance 2021). Evidence from the included studies suggests that AI may support, but not fully replace, aspects of human connectedness within therapeutic care (Garcia et al. 2025), and co‐design approaches identify relational priorities (Wilson et al. 2024). Early work by Louie et al. (2014) provided foundational evidence on the acceptance of socially assistive robots among older adults, emphasising human connectedness as a vital concern, while Abou Hashish's (2025) concept analysis on digital empathy offers cautious optimism regarding AI's potential to enhance therapeutic bonds in nursing care. Although these two papers were not explicitly focused on mental health, core concepts such as therapeutic bonds and considerations around older‐adult cognitive decline transfer into all areas of nursing, including but not limited to mental health contexts (Abou Hashish 2025; Louie et al. 2014). Moreover, Barrera et al.'s (2020) work extends Louie et al.'s (Louie et al. 2014) early evidence of patient acceptance of technology‐mediated observation by demonstrating, in an acute psychiatric ward, that continuous sensor‐based monitoring is not only tolerated but safely embedded into routine care. Yet this success with passive monitoring contrasts with growing concerns over interactive chatbots, where anecdotal evidence points to increasing reliance with negative outcomes (Gawne 2026). This contrast may indicate greater complexity when AI is used in forms of interaction intended to simulate human relational engagement. Nevertheless, critical perspectives such as Rahsepar Meadi et al. (2025) highlight the difficulty of AI *truly* embodying core therapeutic qualities such as empathy and humanness. This tension reflects McCormack and McCance's (2021) assertion that person‐centred care is fundamentally rooted in human values, shared decision‐making, and the distinctive ‘attributes of the nurse’, qualities that AI cannot replicate mechanistically (Bond, Usher, et al. 2025). These are evidenced in evidence‐based mental health nursing communication skills like active listening, cultural sensitivity, and rapport‐building to explore lived experiences (Bond, Hui, et al. 2024). While AI may augment some aspects of therapeutic interaction, uncertainty remains regarding its capacity to replicate the relational qualities central to person‐centred mental health nursing (Bond 2025). These findings also raise broader ethical questions regarding the extent to which AI can align with person‐centred principles in highly relational areas of practice such as mental health nursing (Bond 2025). Hence, chatbots should be used with caution, particularly in clinical contexts requiring nuance and empathy, with careful attention to how they are implemented and the necessity for human oversight.

The reviewed studies highlighted trust and ethics as particularly significant conditions for AI's alignment with person‐centred care values. The importance of maintaining transparency was noted, safeguarding privacy, and ensuring collaborative co‐development among clinicians and patients (Rogan et al. 2024; Tuncer and Duman 2025). These conditions resonate with ethical frameworks and the call for human oversight in AI decision‐making (Rahsepar Meadi et al. 2025). However, the risks identified in predictive applications of AI, such as suicide or violence risk assessments (Hu et al. 2023; Qiao et al. 2025), intensify these ethical dilemmas, as they expose the potential for AI tools to reinforce power imbalances or cause harm through misjudgement or coercion. Without robust governance and oversight, AI applications may create tensions with the principles of dignity and autonomy central to person‐centred care (WHO 2021). Trust emerged across the reviewed studies as an important consideration influencing the acceptability and implementation of AI within person‐centred mental health care. Drawing on Botsman's (2017) typology of trust: institutional (organisational reliability), interpersonal (human relationships), and distributed (platform/algorithmic systems), the integration of AI in mental health nursing may alter how trust is established and maintained within therapeutic relationships. These issues may be particularly significant in mental health settings, where vulnerability, trauma histories, and experiences such as paranoia can shape engagement with technology‐enabled care (Bond et al. 2024). Furthermore, a collaborative approach to the design of AI innovations, between researchers, practitioners, and people with lived experience was absent from most of the studies reviewed (for further exploration of the co‐design process see Vargas et al. 2025). This absence highlights the importance of participatory approaches to AI development and implementation (Higgins et al. 2024). This supports Milasan and Scott‐Purdy's (2025) call for ‘meaningful conversations’ between nurses, patients, and developers, helping to ensure that AI tools support rather than undermine therapeutic relationships. Accordingly, if AI is to align meaningfully with person‐centred care principles in the future, a participatory approach must be undertaken to ensure evaluations of AI tools and other digital technologies are primed to consider what works, for whom, how it works, and in what contexts (Stacey and Bond 2026). Higgins et al. (2024) offer further nursing‐specific clarity, decomposing trust into reliability (technical accuracy), transparency (explainability), and benevolence (value alignment); dimensions paralleling shared decision‐making and meaningfulness that are central to person‐centred care. They position nurses as ‘trust brokers,’ mediating between algorithmic outputs and patient needs, particularly acute in mental health where risk assessment tools carry life‐or‐death implications. There is also potential for uncertainty regarding how patients interpret and respond to AI‐generated interactions within therapeutic settings (Bond et al. 2022).

The reviewed literature suggests that AI may align more closely with person‐centred care when used to complement rather than replace clinical judgement and therapeutic interaction (Watson et al. 2025). AI‐driven tools that enhance safety without disrupting care (Barrera et al. 2020) and conversational agents combined with clinician support (Palmer et al. 2025) demonstrate that this blended approach may sustain both clinical effectiveness and therapeutic presence. Thus, by capturing patient narratives and complementing symptom monitoring (Alves et al. 2025; Garcia et al. 2025), AI could be used to enrich holistic understandings while maintaining the humanistic premise of mental health care. Although this review highlights the growing potential of AI to support person‐centred care in mental health nursing, it also exposes important gaps that warrant future investigation. Of note, the Stanford Report (2025) highlighted the potentially harmful uses of artificial intelligence among young lonely people; for example, AI companion technologies may potentially have unintended consequences for social connection and emotional wellbeing. Clearly, more research is needed to evaluate how AI tools might be meaningfully integrated into therapeutic relationships without displacing the human qualities central to person‐centred mental health nursing (Bond 2025).

Varying methodologies were noted across the studies reviewed, which highlights diverse research approaches to studying the use of AI mental healthcare. However, as Wilson et al. (2024) illustrate, there is a critical need for co‐design methodologies that systematically embed patient/carer/lived experience into technology development. Ultimately, these methodological differences emphasise the multifaceted nature of research into AI while revealing co‐design's unique value in translating quantitative acceptance data and qualitative relational insights into person‐centred technologies that complement (but never supersede) the essential human qualities of trust and empathy required for truly person‐centred nursing (Bond 2025; Watson et al. 2025; Moreno‐Poyato et al. 2021).

For future studies, a longitudinal approach would be beneficial for examining the sustained impact of AI‐enabled interventions on patient outcomes, engagement, and trust over time. Comparative research across diverse cultural and service contexts would also help to clarify whether AI applications reduce inequities or inadvertently reinforce existing disparities. Further methodological work is also required to develop frameworks for capturing patient narratives and relational depth within digital platforms, ensuring that AI supports, rather than diminishes, relational aspects of care such as empathy, trust, compassion, and shared decision‐making (Bond et al. 2024).

### 5.1 Strengths and Limitations

This review has synthesised a diverse body of literature on artificial intelligence (AI) applications in mental health nursing, incorporating studies across varied populations, geographies, sample sizes, and methodological designs. A key strength lies in the comprehensive understanding gained by combining quantitative and qualitative data, which allows for a nuanced exploration of AI's multifaceted role, from clinical effectiveness to ethical considerations and person‐centred care. The inclusion of studies in Sweden, Singapore, Türkiye, Canada, UK, China, Ireland, Thailand, and other European contexts enhances the review's global relevance and broadens the applicability of findings across different health systems and cultural contexts. The wide variety of populations studied, such as adolescents receiving internet‐based cognitive behavioural therapy, older adults engaging with AI chatbots, psychiatric inpatients, mental health professionals, and individuals living with dementia and their carers, provides an inclusive picture of the impact of AI across the lifespan and care spectrum. Methodologically, this review benefits from integrating diverse designs including qualitative interviews, non‐randomised experimental studies, cross‐sectional analyses, and mixed‐method approaches. This mutually informing combination of data deepens insight into both objective outcomes and subjective experiences, such as ethical concerns and relational dynamics with AI. A targeted database strategy maximised mental health nursing literature retrieval but excluded broader databases, which may have potentially missed relevant literature related to AI applications, primarily indexed outside nursing literature. Additionally, the heterogeneity of the studies included presents limitations. The broad variability in study designs, sample sizes (ranging from small qualitative samples to larger observational cohorts), and cultural contexts makes direct comparisons difficult and limits generalisability of specific findings. Smaller qualitative samples may also limit the transferability of certain insights to wider populations. Moreover, the diversity of measures and AI applications used across the studies makes it impossible to synthesise findings into cohesive, universally applicable conclusions.

## 6 Conclusion

AI offers potential to enhance person‐centred mental health nursing by increasing access, supporting symptom monitoring, and empowering populations who may be underserved. Yet these benefits are contingent on its integration being firmly grounded in the core humanistic values of authentic empathy, trust, and relational depth that remain central to effective care. Without such grounding, AI risks reinforcing surveillance‐oriented practices, efficiency‐driven care models, and forms of depersonalisation that sit in tension with recovery‐oriented mental health nursing. More fundamentally, however, it remains uncertain whether AI can fully align with person‐centred principles in relational fields such as mental health nursing, or whether its limitations impose inherent constraints on its ethical application in practice.

AI should complement rather than replace the nurse's therapeutic role, supported by ethical oversight, transparency, and ongoing training. Mental health nurses are therefore not merely end‐users of AI technologies, but key ethical actors and trust brokers who mediate between algorithmic systems and the lived experiences of patients. Thus, mental health nurses must seek to recognise AI's limits and prioritise models that uphold dignity, shared decision‐making, and human connection. They must also continue to advocate for the development and implementation of AI‐driven platforms that are demonstrably beneficial for patients, including active involvement in design, evaluation, and governance processes to ensure that person‐centred values are not subordinated to technical efficiency or organisational imperatives. Within these constraints, AI‐driven applications may offer opportunities to support more holistic and responsive care; however, their role in sustaining the principles of person‐centred practice remains dependent on ongoing critical, ethical, and relational scrutiny.

## 7 Relevance for Clinical Practice

AI may create new opportunities for mental health nursing, from supporting more accurate assessments to helping track changes in symptoms and tailoring care to individual needs. However, for these tools to be meaningfully integrated into practice, it is important that nurses and patients are actively involved in how they are developed and used, ensuring AI supports rather than constrains clinical judgement.

Nurses need to feel confident that AI can be used safely and ethically, yet there remains potential for unintended harm if these technologies are not carefully implemented, monitored, and critically appraised in practice. Protecting patient privacy, ensuring transparency in how systems operate, and maintaining trust remain important considerations, alongside the need for ongoing training and support for the workforce.

Above all, AI appears most appropriately positioned as a complement to, not a replacement for, the therapeutic relationships and empathy that underpin mental health nursing. While AI may support more responsive and efficient care delivery, its use requires continued ethical scrutiny to ensure that relational, person‐centred values are not diminished within increasingly digital models of care. This review highlights that the integration of AI into mental health nursing is not solely a technical or operational issue, but also a relational, and ethical one. As AI‐enabled technologies become more embedded within mental health services, careful attention will be required to ensure that efficiency and innovation do not overshadow the relational foundations of therapeutic care. The findings of this review suggest that the future role of AI in mental health nursing will depend not only on technological capability, but also on the extent to which these systems can be implemented in ways that preserve human connection and person‐centred care.

## Funding

The authors have nothing to report.

## Conflicts of Interest

The authors declare no conflicts of interest.

## Data Availability

Data sharing not applicable to this article as no datasets were generated or analysed during the current study.

## Appendix A Database Search Strategies

Complete search strategies by database:

**Table:**

Database	Search strategy (as executed)	Limits	Records retrieved
Scopus	TITLE‐ABS‐KEY(mental health nurs* OR psychiatric nurs*) AND TITLE‐ABS‐KEY(artificial intelligence OR AI OR machine learning OR deep learning OR ChatGPT)	Article Humans English 01/07/2018–01/07/2025	104
CINAHL	(mental health nurs* OR psychiatric nurs* OR psych nurs* OR mental health nurse* OR psychiatric nurse* OR psych nurse*) AND (artificial intelligence OR AI OR machine learning OR deep learning OR ChatGPT) NOT student*	English Peer‐reviewed 01/07/2018–01/07/2025	41
APA PsycInfo	(mental health nurs* OR psychiatric nurs* OR psych nurs* OR mental health nurse* OR psychiatric nurse* OR psych nurse*) AND (artificial intelligence OR AI OR machine learning OR deep learning OR ChatGPT) NOT student*	English Peer‐reviewed 01/07/2018–01/07/2025	15
